# A new species of Diaspididae, *Megacanthaspisguiyangensis* (Hemiptera, Coccomorpha) from China

**DOI:** 10.3897/zookeys.858.35363

**Published:** 2019-07-01

**Authors:** Minmin Niu, Jinian Feng

**Affiliations:** 1 Key Laboratory of Plant Protection Resources and Pest Management, Ministry of Education, Entomological Museum, College of Plant Protection, Northwest A&F University, Yangling, Shaanxi Province, 712100, China Northwest A&F University Yangling China

**Keywords:** Armoured scale, diagnosis, illustration, key, taxonomy

## Abstract

A new species of armoured scale insect, *Megacanthaspisguiyangensis***sp. nov.** is described and illustrated. The new species is morphologically similar to *M.hangzhouensis*. *Megacanthaspisguiyangenis* infests leaves of *Oligostachyumlubricum* in China. A key to all eight species of *Megacanthaspis* now known is provided.

## Introduction

Armoured scale insects (Hemiptera: Coccomorpha: Diaspididae), are the largest family of the Coccoidea, and have a worldwide distribution, including 426 genera and 2624 species currently identified (García Morales et al. 2016). The morphology of adult females is extremely reduced: no legs, antennae reduced to unsegmented tubercles, head, thorax and abdomen are fused, and these adults are wingless (Balachowsky 1948; Takagi 1993; Andersen et al. 2010; Henderson 2011).

The genus *Megacanthaspis* Takagi, 1961 belongs to the tribe Diaspidini. The genus was originally established by Takagi (1961) with *Megacanthaspisactinodaphnes* Takagi, 1961 designated as the type species. The genus is currently composed of seven species (García Morales et al. 2016).

The genus *Megacanthaspis* is distributed in China, Japan, and Nepal (García Morales et al. 2016). Takagi (1961) collected the type species of this genus in Japan. Later he described another species, *M.litseae*, from Taiwan, China (Takagi, 1970), and added two new species (*M.langtangana* and *M.leucaspis*) from Japan (Takagi, 1981). Takagi (1981) also transferred the species *Nanmuaspisphoebia* Tang, collected in China (Tang, 1977), into *Megacanthaspis*. Most recently Wei (2012) recorded two new species of this genus (*M.hangzhouensis* and *M.hainanensis*) from China.

A new species of *Megacanthaspis* was discovered in China and is described and illustrated in this work. This discovery raises the number of species recorded in the genus to eight, five of which have been reported from China. A key to all species of the genus *Megacanthaspis* is provided.

## Materials and methods

Samples of plants infested by the new species described in this study were collected in Guiyang City (Guizhou Province, China). Permanent slide mounts of adult females from the samples collected were slide-mounted using the protocol described by Henderson (2011).

Illustrations of adult female of the new species were drawn from the slide-mounted specimens, showing an overview of the dorsum on the left side and the venter on the right; enlarged details of the significant features are illustrated but not drawn in direct proportion to each other.

All specimens of the new species, *Megacanthaspisguiyangensis*, were deposited in the Entomological Museum, Northwest A&F University, Yangling, Shaanxi, China (**NWAFU**).

### Taxonomy

#### Megacanthaspis

Taxon classificationAnimaliaHemipteraDiaspididae

Takagi, 1961

Megacanthaspis Takagi, 1961: 97.

##### Type species.

*Megacanthaspisactinodaphnes* Takagi by monotype and original designation.

##### Generic diagnosis.

**Adult female**. Body elongate and slender, with metathorax and free abdominal segments not strongly produced; derm membranous. Each antenna with a long seta. Anterior spiracles with disc pores, and posterior spiracles of some species also with disc pores. Gland tubercles present caudad of anterior spiracles, laterocaudad of posterior spiracles and submarginally on 1–3 anterior abdominal segments. Pygidium rounded along posterior margin, all species without lobes, some species with a marginal series of serrate processes or plates. Marginal gland spines present on the abdomen, each with one or more microducts. Dorsal macroducts present on abdomen and arranged in segmental rows but not in a well-defined series. Ventral ducts are the same size or smaller than dorsal ducts. Anal opening situated in the centre of pygidium. Perivulvar pores with five groups or connected to form an arc.

##### Remarks.

The genus *Megacanthaspis*, like other groups such as *Thysanaspis* and *Pygalataspis*, has non-glanduliferous plates that are well developed but does not have distinct lobes. *Thysanaspis* and *Pygalataspis* have no gland spines. Members of the genera *Megacanthaspis*, *Kuwanaspis*, and *Nikkoaspis* all have plates and gland spines.

#### Megacanthaspis guiyangensis sp. nov.

Taxon classificationAnimaliaHemipteraDiaspididae

http://zoobank.org/1B30EFDA-374A-4EC7-B01E-15E325415428

##### Material studied.

Holotype female: CHINA, Guizhou Province, Guiyang city, 26°24'35"N; 106°40'13"E. Collected on *Oligostachyumlubricum* leaves by Niu & Wei, 21.vii.2015, fist specimen from the left end of a row of 5 adult females, clearly indicated on the slide label (NWAFU).

Paratypes: 59 specimens, same data as holotype (at, 1 slide with 1 adult female, 1 slide with 2 adult females, 1 slide with 3 adult females, 4 slides each with 4 adult females, 5 slides each with 5 adult females, 2 slides each with 6 adult females (NWAFU).

##### Description.

**Figures 1–8.**
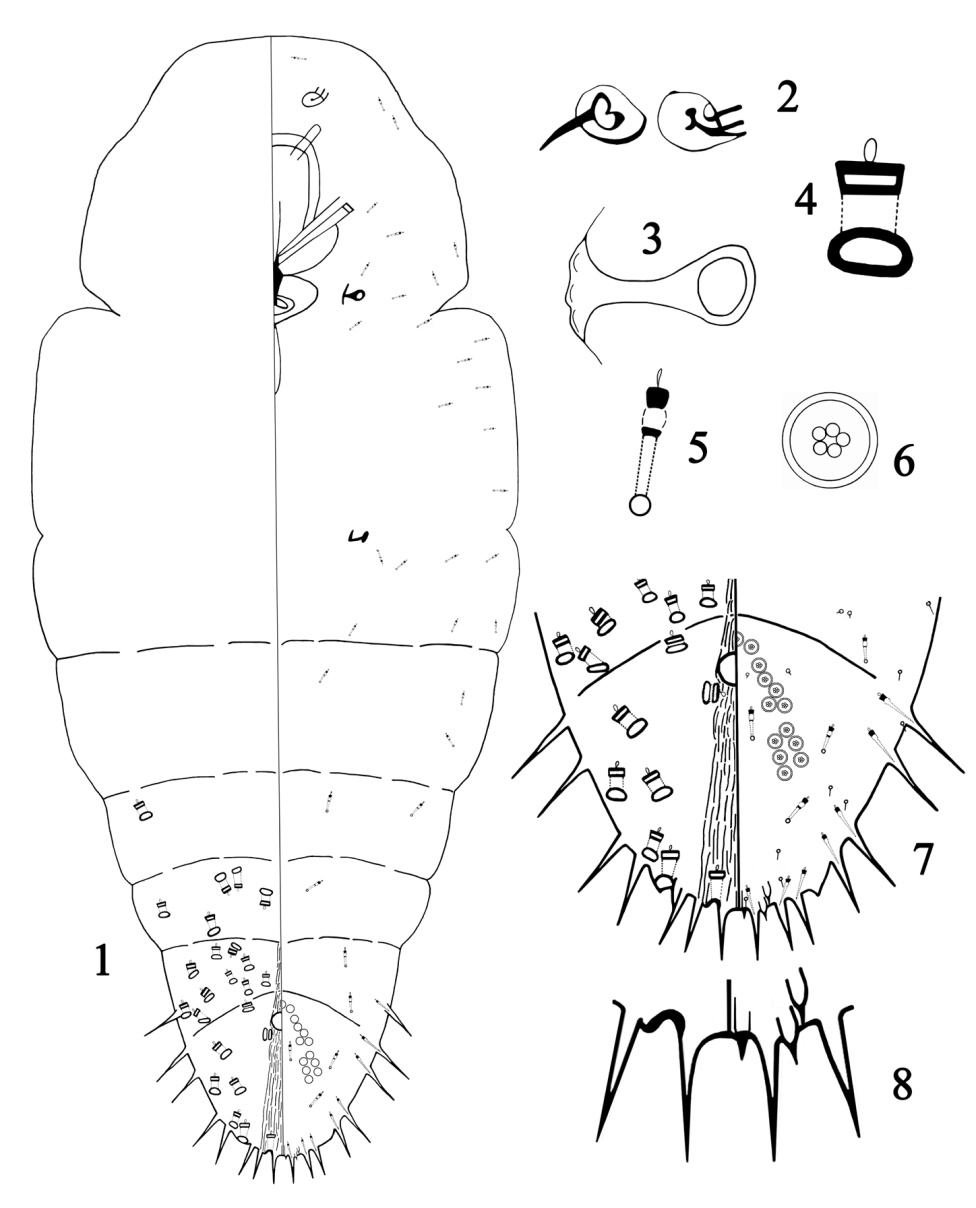
*Megacanthaspisguiyangensis* sp. nov., adult female **1** body **2** antennae **3** anterior spiracle **4** detail of dorsal gland duct **5** ventral microducts **6** perivulvar pore **7** pygidium **8** margin of pygidium (sharp marginal processes).

**Adult female.** (Figs 1–8) Body outline oblong fusiform, with indistinct segmentation. Each antenna with a long seta and a tubercle. Anterior and posterior spiracles without disc pores. The pygidium with sharp marginal processes on abdominal segments VII–VIII: 2 on each side of abdominal segment VII, 2 on abdominal segment VIII between the marginal gland spines (Fig. 8). Marginal gland spines each associated with one microduct, present on abdominal segments V–VIII: one pair, widely separated, on abdominal segment VIII and two pairs on abdominal segments V–VII. Some individuals have one pair of marginal gland spines on abdominal segment IV. Gland tubercles absent. Dorsal macroducts arranged in irregular rows on abdominal segments II–VIII and numbering approximately 23–50 on each side; Marginal macroducts arranged one on each side of abdominal segment VII, and absent on abdominal segment VIII between the gland spines. Ventral microducts smaller than dorsal macroducts, scattered loosely on the cephalothorax and abdomen. Anus rounded, located near the centre of the pygidium. Five groups of perivulvar pores form arcs: 3–5 in the median group, 5–8 in each laterocephalic group, and 4–8 in each laterocaudal group.

##### Diagnosis.

*Megacanthaspisguiyangensis* sp. nov. resembles *M.hangzhouensis* (Wei & Feng, 2012) in body outline, absence of gland tubercles and with 1 microduct on each of the marginal gland spines. The important differences between the pygidia of the two species are shown in Table 1.

**Table 1.** Morphological differences between the pygidia of *M.guiyangensis* sp. nov. and *M.hangzhouensis*.

**Pygidium character state**	***M.guiyangensis* sp. nov.**	*** M. hangzhouensis ***
Marginal processes	present	absent
Pairs of gland spines on abdominal segment VII	2	1
Number of dorsal macroducts on each side of abdomen	23-50	about 17

##### Host.

*Oligostachyumlubricum* (Poaceae).

##### Etymology.

Named after Guiyang, the type locality.

##### Distribution.

China (Guizhou).

### Key to adult female *Megacanthaspis* Takagi

**Table:** 

1	Marginal gland spines each with a single microduct	**2**
–	Marginal gland spines each with 2 or more microducts	**4**
2	Marginal processes absent	***M.hangzhouensis* Wei**
–	Marginal processes present	**3**
3	Marginal sharp processes on abdominal segment VII–VIII.	***M.guiyangensis* sp. nov.**
–	Marginal serrate processes on abdominal segment V and VI as well as VII–VIII	***M.leucaspis* Takagi**
4	Gland spines on abdominal segment VIII close together	**5**
–	Gland spines on abdominal segment VIII separated	**6**
5	Marginal gland spines present on segment II	***M.langtangana* Takagi**
–	Marginal gland spines absent from segment II	***M.actinodaphnes* Takagi**
6	With a macroduct between median gland spines on abdominal segment VIII	***M.hainanensis* Wei**
–	Without macroduct between median gland spines on abdominal segment VIII	**7**
7	Marginal serrate processes present on abdominal segment VI as well as on segments VII–VIII	***M.phoebia* (Tang)**
–	Marginal serrate processes present on abdominal segments VII–VIII	***M.litseae* Takagi**

## Supplementary Material

XML Treatment for Megacanthaspis

XML Treatment for Megacanthaspis guiyangensis
